# A Multicentre Molecular Analysis of Hepatitis B and Blood-Borne Virus Coinfections in Viet Nam

**DOI:** 10.1371/journal.pone.0039027

**Published:** 2012-06-13

**Authors:** Linda Dunford, Michael J. Carr, Jonathan Dean, Linh Thuy Nguyen, Thu Hong Ta Thi, Binh Thanh Nguyen, Jeff Connell, Suzie Coughlan, Hien Tran Nguyen, William W. Hall, Lan Anh Nguyen Thi

**Affiliations:** 1 Ireland Viet Nam Blood-Borne Virus Initiative (IVVI), Dublin, Ireland and Ha Noi, Viet Nam; 2 National Virus Reference Laboratory, University College Dublin, Dublin, Ireland; 3 Laboratory for Molecular Diagnostics, National Institute of Hygiene and Epidemiology, Ha Noi, Viet Nam; Drexel University College of Medicine, United States of America

## Abstract

Hepatitis B (HBV) infection is endemic in Viet Nam, with up to 8.4 million individuals estimated to be chronically infected. We describe results of a large, multicentre seroepidemiological and molecular study of the prevalence of HBV infection and blood-borne viral coinfections in Viet Nam. Individuals with varying risk factors for infection (n = 8654) were recruited from five centres; Ha Noi, Hai Phong, Da Nang, Khanh Hoa and Can Tho. A mean prevalence rate of 10.7% was observed and levels of HBsAg were significantly higher in injecting drug users (IDUs) (17.4%, n = 174/1000) and dialysis patients (14.3%, n = 82/575) than in lower-risk groups (9.4%; *p*<0.001). Coinfection with HIV was seen in 28% of HBV-infected IDUs (n = 49/174) and 15.2% of commercial sex workers (CSWs; n = 15/99). HCV infection was present in 89.8% of the HBV-HIV coinfected IDUs (n = 44/49) and 40% of HBV-HIV coinfected CSWs (n = 16/40). Anti-HDV was detected in 10.7% (n = 34/318) of HBsAg positive individuals. Phylogenetic analysis of HBV *S* gene (n = 187) showed a predominance of genotype B4 (82.6%); genotypes C1 (14.6%), B2 (2.7%) and C5 (0.5%) were also identified. The precore mutation G1896A was identified in 35% of all specimens, and was more frequently observed in genotype B (41%) than genotype C (3%; *p*<0.0001). In the immunodominant ‘a’ region of the surface gene, point mutations were identified in 31% (n = 58/187) of sequences, and 2.2% (n = 4/187) and 5.3% (n = 10/187) specimens contained the major vaccine escape mutations G145A/R and P120L/Q/S/T, respectively. 368 HBsAg positive individuals were genotyped for the *IL28B* SNP rs12979860 and no significant association between the *IL28B* SNP and clearance of HBsAg, HBV viral load or HBeAg was observed. This study confirms the high prevalence of HBV infection in Viet Nam and also highlights the significant levels of blood-borne virus coinfections, which have important implications for hepatitis-related morbidity and development of effective management strategies.

## Introduction

The World Health Organisation (WHO) has estimated that over 350 million people worldwide are chronically infected with Hepatitis B virus (HBV) which results in approximately 600,000 HBV related deaths each year, primarily from cirrhosis and hepatocellular carcinoma (HCC) [1]. In geographic regions with low HBV endemicity, the virus is normally acquired in adulthood through horizontal routes of transmission, which include high risk sexual behaviour, receipt of blood products, blood-blood contact or injecting drug use. In contrast, in countries with intermediate and high endemicity, HBV is primarily acquired by vertical transmission perinatally or early in childhood [2], [3].

There are approximately 8.4 million individuals chronically infected with HBV in Viet Nam and it was estimated that in 2005 this resulted in 23,300 HBV-related mortalities [4]. Previous reports of HBV surface antigen (HBsAg) prevalence have suggested levels as high as 15–20% in the general population [5]–[13]; with reported prevalences in neonates, children and adolescents of 12%, 18% and 20%, respectively [10]. Universal HBV vaccination was introduced in Viet Nam in 2003; however, despite this, mathematical models have predicted that Viet Nam in the future faces an enormous burden of HBV-related liver disease [4].

Eight HBV genotypes (designated A through H) and several subgenotypes have been identified to date, and these have distinct geographic distributions [14]. In addition, a complex recombinant of genotypes C, A and G, first described in Viet Nam in 2000 [15], has been proposed to be a new genotype designated “I” [16]. However this remains controversial as the mean genetic divergence of the Vietnamese HBV A/C/G recombinant is <8% from genotype C across the entire genome and is thus considered as having arisen from intragenotypic, not intergenotypic, divergence [17]. This variant was subsequently identified in Laos [18], China [19], [20], India [21] and in emigrants and children adopted from Viet Nam and living in France and Canada [22], [23]. An additional genotype “J” has also been proposed for a virus isolated from one Japanese male [24].

Human Immunodeficiency Virus (HIV) and Hepatitis C virus (HCV) coinfections in individuals with chronic HBV infection are increasingly common due to shared routes of transmission. Globally, it is estimated that 4 million chronically HBV-infected individuals are coinfected with HIV [25]. Limited information is available on the prevalence of coinfections in Southeast Asia because of a lack of routine screening. Zhou *et al.* reported a large serosurvey of HIV, HBV and HCV infection among injecting drug users (IDUs) along the Chinese-Myanmar border and indentified 20% HIV-HBV coinfections and 11% HIV-HBV coinfections in Chinese and Burmese IDUs, respectively, with a comparable prevalence of HIV-HBV-HCV triple infections (19% and 10%, respectively) in each population [26]. Unlike HBV, the HIV epidemic in Viet Nam is concentrated in high risk groups, such as IDUs, commercial sex workers (CSWs) and men who have sex with men (MSM) [27], [28]. A study from Hai Phong in Northern Viet Nam reported a HBsAg prevalence of 10.3% in HIV-infected individuals [13]. In industrialised countries, the introduction of highly active antiretroviral therapy (HAART) has lead to a significant decline in deaths attributable to HIV; however, liver disease has since emerged as a leading cause of morbidity and mortality in HIV infected individuals coinfected with HBV and HCV [29], [30]. Management of HBV in HIV-infected individuals is also complicated by the emergence of drug resistant viruses and HAART-associated hepatotoxicity. The use of antiretroviral drugs may result in the emergence of cross-resistance mutations in the HBV *pol* gene. Specifically, it has been reported, that in HIV-HBV coinfected patients treated with lamivudine, resistance mutations within the YMDD motif in the HBV polymerase reverse transcriptase (RT) domain occur at a rate of approximately 25% per year, compared to approximately 16% in HBV monoinfected patients [31], [32].

**Figure 1:**
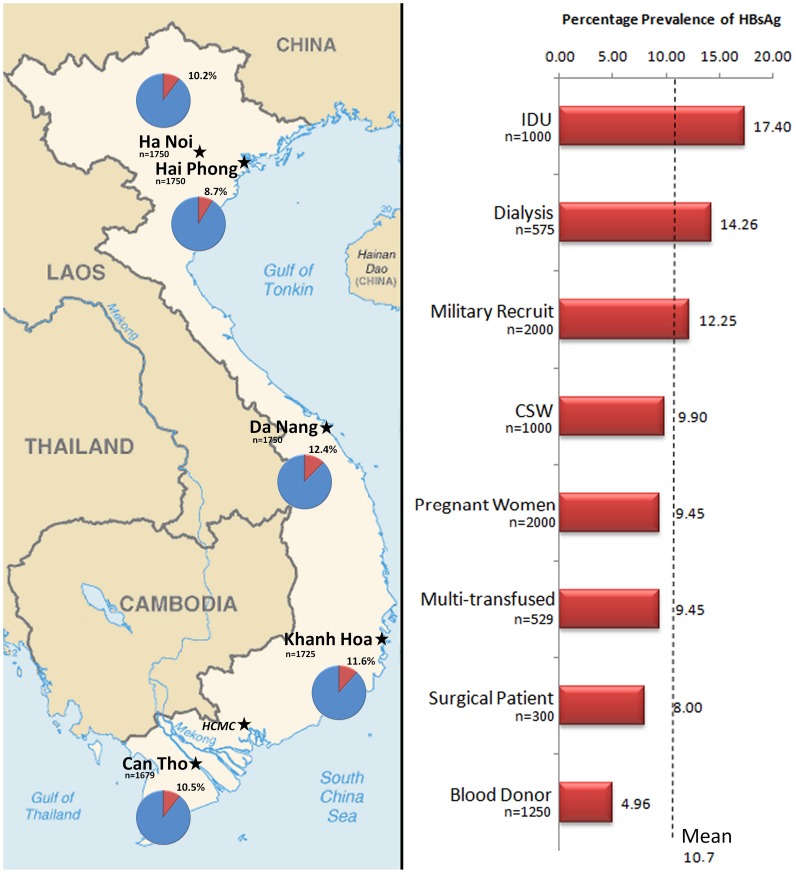
Map of Viet Nam Depicting the Prevalence of HBsAg in 5 Regions. The map depicts the percentage HBsAg positives in Ha Noi, Hai Phong, Da Nang, Khanh Hoa and Can Tho. To the right is a graph depicting the prevalence of HBsAg in each of the study groups in the 5 study sites in Viet Nam.

Hepatitis Delta virus (HDV) is a small, defective RNA virus that utilises HBsAg to produce infectious particles and can only replicate in individuals already infected with HBV [33], [34]. Globally, an estimated 18 million (ca. 5%) of the 350 million chronic HBV carriers have serological evidence of prior exposure to HDV and HBV-HDV coinfection has been reported to have a more severe clinical course than HBV monoinfected individuals [35]–[39]. The incidence of HDV infection appears to be decreasing worldwide due to the impact of HBV vaccination programmes. Typically, HDV prevalence is highest in regions where HBV is endemic [34]; however, previous studies have reported very low or undetectable levels of HDV infection in Viet Nam [11], [40].

**Figure 2:**
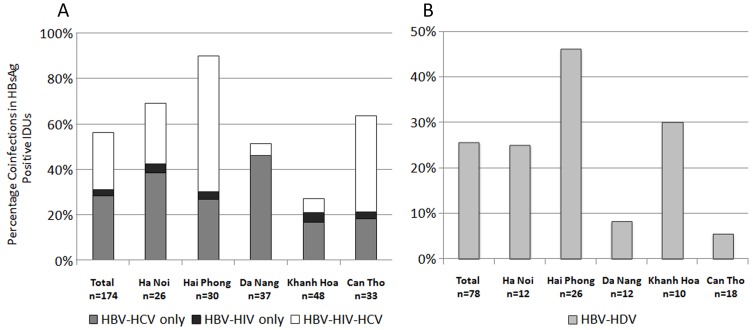
Percentage of Blood-Borne Viral Coinfections in HBsAg Positive Intravenous Drug Users. **“**A” represents the proportion of HBsAg positive IDUs (n = 174) also testing positive for HIV Ab/Ag and HCV Ab/Ag (n = 98) and “B” represents the proportion of HBsAg positive IDUs (n = 78) testing positive for HDV (n = 20).

Several viral factors, including genotype and specific viral mutations, have been documented to influence the clinical outcome of HBV infection. Among these, HBV genotype is not only a predictor of clinical outcome but has also been associated with response to interferon treatment [41]. Although there is a well established predominance of HBV genotypes B and C in Southeast Asia, including Viet Nam, previous reports suggest that there may be significant genetic heterogeneity at the subgenotype level; HBV subgenotypes B2, B4, C1 and C5 have been reported in Viet Nam with subgenotypes B4 and C1 predominating [13], [40], [42]–[46]. In addition, the putative HBV genotype I has been reported to account for 1% of circulating virus in Viet Nam [46]. Emerging data has shown that HBV viral load and naturally occurring mutant strains may be closely associated with progression to severe liver disease [41]. For example, genotype C has a higher frequency of mutations in the basal core promoter (*BCP*) and deletions in the *preS* region of the genome and is associated with higher viral loads than genotype B [41]. HBV genotype C infection has been associated with a decreased rate of response to interferon-α therapy compared with genotype B [41]. Selection pressures including vaccination, antiviral therapy and host immune response may result in the emergence of viral variants which are associated with progression to more severe liver disease [47].

**Figure 3:**
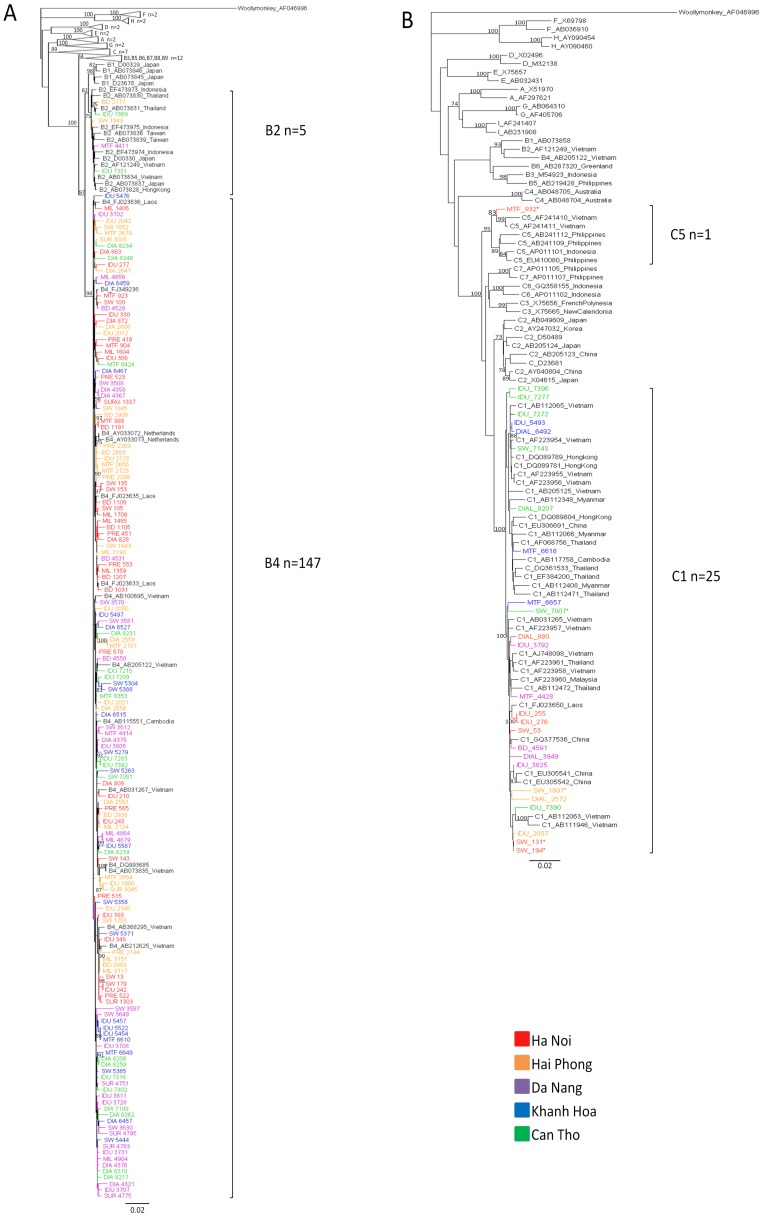
Phylogenetic Analysis of 1070 nt Region of the HBV *Pol* Gene of Specimens Identified in the IVVI Study. The taxons and branches of Vietnamese specimens whose sequences were identified in this study are coloured by sample location from Ha Noi (red), Hai Phong (orange), Da Nang (purple), Khanh Hoa (blue) and Can Tho (green). Sequence taxons are coded by population group including intravenous drug users (IDU), sex workers (SW), dialysis patients (DIAL), multiply transfused patients (MTF), military recruits (MIL), pregnant women (PRE), blood donors (BD) and elective surgery patients (SUR). Figure 2A represents a phylogenetic analysis of genotype B sequences, including 152 sequences from HBV subtype B described in this study, with 64 reference sequences (labelled with their Genbank accession numbers and country of isolation). Branches of reference sequences from groups A, C, D, E, F, G and H are collapsed. The newly described putative subgenotype B6–B9 sequences were included and branches have been collapsed. Figure 2B represents 26 sequences from HBV subtype C described in this study, with 73 reference sequences. Brackets denote the subgenotypes identified in this study and the number of IVVI sequences in these groups. Genbank accession numbers for the study sequences are JQ281112–JQ281258 and JQ281468–JQ281471.

Recently, genome wide association studies have highlighted the impact of host genetics on the outcome and impact of viral infection. Genetic variation adjacent to the type III interferon gene, interleukin-28B (IL28B) on chromosome 19q13 is strongly associated with both treatment-induced and natural clearance of HCV; in contrast, the effect on HBV infection is unclear [48]–[50]. However, recent studies have reported an association between the *IL28B* locus and response to interferon- α therapy [51], [52].

Here, we describe the results of a large-scale, national study, estimating the prevalence of hepatitis B virus and blood-borne virus coinfections in different regions and different risk groups in Viet Nam.

## Materials and Methods

### Ethics Statement

Ethical approval for the study was obtained from the National Institute of Hygiene and Epidemiology (NIHE) in Ha Noi. All specimens and survey information were obtained with informed written consent and subsequently anonymised.

**Table 1:** Correlation of HBV Viral Load and Genotype in HBeAg Positive and Negative Individuals (n = 217).

	HBeAg positive	HBeAg negative	*p –* value	Statistical Method
n =	87	127		
**Mean Age (yrs ± SD, range)**	24.1±7.4 (16–55)	32.7±13.3 (18–79)	<0.0001	2 tailed t test
**% Male**	58.6%	42.5%	0.02	*χ* ^2^
***HBV DNA (Log_10_ IU/ml)***				
**Mean Viral Load**	7.58±1.34 (2.45–8.9)	3.32±1.65 (0.15–8.36)	<0.0001	2 tailed t test
**DNA undetectable (%)**	4.6%	19.7%	0.002	*χ* ^2^
**≤2,000**	2.3%	44.9%	<0.0001	*χ* ^2^
**2,001–20,000**	2.3%	20.5%	<0.0001	*χ* ^2^
**20,001–200,000**	1.1%	5.5%	0.098	*χ* ^2^
**200,001–2,000,000**	8.0%	4.7%	0.499	*χ* ^2^
**>2,000,001**	81.6%	4.7%	<0.0001	*χ* ^2^

*HBV Genotype*	n = 64	n = 82		
**B**	79.7%	87.8%	0.285	*χ* ^2^
**C**	14.1%	8.5%		

All individuals in this cohort tested positive for HBsAg and serologically negative for HIV, HCV and HDV.

The study is part of the Ireland Viet Nam Blood-Borne Virus Initiative (IVVI) which is a partnership between the National Virus Reference Laboratory (NVRL) in Dublin, Ireland and the National Institute of Hygiene and Epidemiology (NIHE) in Ha Noi, Viet Nam. In all, 8654 specimens were serologically tested for HIV, HBV and HCV infection. The study involved eight population groups including commercial sex workers (CSWs), injecting drug users (IDUs), blood donors, military recruits, pregnant women, dialysis patients, elective surgery patients and recipients of multiple blood transfusions. Paired serum and plasma specimens were collected in 2008 and 2009, along with detailed demographic information, from five centres throughout Viet Nam: Ha Noi (n = 1750) and Hai Phong (n = 1750) in the North, Da Nang (n = 1750) in the Central region and Khanh Hoa (n = 1725) and Can Tho (n = 1679) in the South.

**Figure 4:**
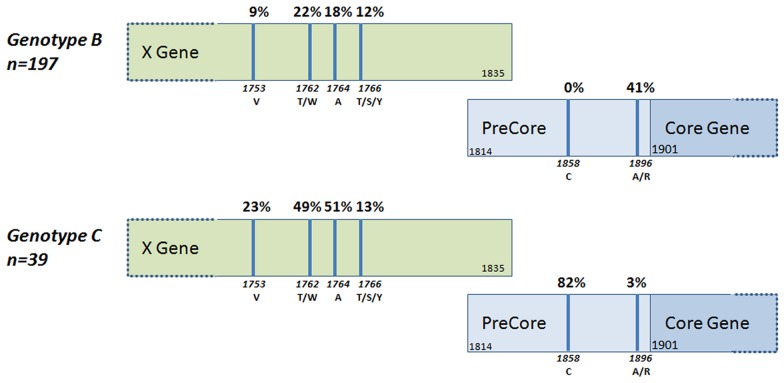
Mutations in the Basal Core Promoter and PreCore Regions of the HBV Genome in Genotype B and C Viruses. The precore stop mutation G1896A was identified in 35% (n = 82/236) of all samples and varied significantly by viral genotype, with a higher occurrence in genotype B (41%) compared to Genotype C (3%) (*p*<0.001). In contrast, the basal core promoter mutations A1762T and G1764A were detected more frequently in genotype C at 49% and 51% respectively, compared to only 22% and 18% in genotype B (*p*<0.0001). The mutation T1858C was identified only in genotype C viruses.

### Viral Serology

All specimens were tested using commercially available EIAs for HBsAg using Murex HBsAg version 3 (Abbott Laboratories, IL, USA), for HIV antibody and antigen using the Genscreen HIV Ab-Ag serological screening kit (Bio-Rad Laboratories, CA, USA) and for HCV using the MONOLISA Ag/Ab HCV Ultra (Bio-Rad Laboratories, CA, USA). A representative subset (n = 372) of HBsAg positive specimens, collected from high and low risk groups in the five study sites, were chosen for additional serological markers and detailed molecular analysis. Specimens were tested for the presence of HBeAg (n = 356) and, if negative, for anti-HBe (n = 208) using ETI-EBK-PLUS (HBeAg) and ETI-AB-EBK PLUS (anti-HBe; DiaSorin, Saluggia, Italy). 276 samples (including all HBeAg positive samples) were tested for HBc IgM using ETI-CORE-IgMK PLUS (DiaSorin, Saluggia, Italy). A selection of 110 HBsAg negative samples were screened for HBV total core (anti-HBc) using the MONOLISA anti-HBc Assay (Bio-Rad Laboratories, CA, USA). In addition specimens (n = 319) were tested for evidence of infection with delta virus using ETI-AB-Delta-K2 (anti-HDV) and positives were tested for IgM anti-HDV with ETI-DELTAK-IGMK-2 assay (DiaSorin, Saluggia, Italy; n = 319).

**Table 2:** Association of *IL28B* Genetic Variation at rs12979860 with HBV Viral Load and Serology.

	CC	CT + TT	*p –* value	OR (95% CI)
**HBsAg positive, n = 214**	85.0	15.0	0.498	1.377 (0.5444–3.488)
**HBV negative, n = 53**	88.7	11.3		
**HBeAg positive, n = 87**	92.0	8.0	0.059	2.472 (0.965–6.333)
**HBeAg negative, n = 127**	80.3	19.7		
**HBV DNA ≤2×10^4^ Log_10_ IU/ml, n = 120**	82.5	17.5	0.238	1.601 (0.729–3.514)
**HBV DNA>2×10^4^ Log_10_ IU/ml, n = 94**	88.3	11.7		

All individuals in this cohort were serologically negative for HIV, HCV and HDV.

### Quantitative PCR and Molecular Characterisation

Nucleic acids were extracted from 200 µl of plasma from HBsAg positive (n = 376) and negative specimens (n = 110), using the QIAamp DNA Blood Mini Kit (Qiagen, Crawley, UK) as per the manufacturer's instructions, with a final elution in 50 µl. λ phage DNA (25 pg/sample) was added to the lysis buffer during the extraction as an exogenous internal control for the viral load assay [53].

HBV viral load (VL) was determined using an “in-house” quantitative real-time polymerase chain reaction (qPCR) with serial dilutions of a plasmid-derived HBV DNA standard were used to prepare the standard curve and λ phage DNA co-amplified as an internal control. The PCR reaction was performed with 10 µl of extracted DNA in a 25 µl total reaction volume with the Platinum qPCR Supermix kit (Invitrogen™ Life Technologies, Paisley, UK), and 0.4 µM oligonucleotide primers and 0.2 µM probes targeting a highly conserved region of the HBV *S* gene [54]. The reaction was amplified on an ABI 7500 FAST real-time platform (Applied Biosystems) with an initial 2 min incubation at 50°C, followed by 10 min at 95°C and 45 cycles of 95°C for 15 s and 60°C for 1 min. The assay was calibrated against the WHO 2^nd^ International Reference Standard for HBV DNA (NIBSC: 97/750) and validated with a 95% limit of quantitation of 500 IU/ml plasma (2.69 log_10_ IU/ml) and a linear dynamic range of 5E2–5.4E9 IU/ml (2.69–9.73 log_10_ IU/ml). The assay was validated to ensure high concordance with commercially available HBV DNA platforms, giving R^2^ values of 0.91 and 0.96 when compared to Siemens VERSANT bDNA 3.0 and Roche COBAS® Ampliprep-COBAS TaqMan® HBV quantitiative assays, respectively.

HBV genotype and mutation analyses were determined for specimens with detectable HBV DNA using a hemi-nested PCR targeting a 1.1 kb fragment of the *pol* gene, incorporating partial *preS1* and the entire *preS2* and *S* genes (fragment B), and a nested PCR targeting the basal core promoter/precore region (fragment D) [55], [56] (see Table S1 for all oligonucleotide primers employed in this study). In a subset of specimens (n = 28) the two additional flanking fragments A and C were amplified to obtain whole-genome sequences. Assays for fragments A, B and C were modified from previously published studies [55], [57], [58]. For fragment B, 5 µl of extracted DNA was used with Expand High Fidelity PCR system (Roche Applied Sciences, Mannheim, Germany) in both rounds of a hemi-nested PCR as follows: primers P4mod and P5mod – with a 2 min initial denaturation at 94°C, followed by 10 cycles of 94°C for 15 s, 60°C for 30 s and 72°C for 60 s and a further 20 cycles of 94°C for 15 s, 50°C for 30 s and 72°C for 60 s and a final extension step of 7 min at 72°C. The PCR product was further amplified in a hemi-nested PCR with primers P5mod and P6 using the same cycling conditions with 30 cycles instead of 20 cycles for the second amplification phase. The products were visualised on a 1% TAE agarose gel with 0.01% (v/v) of SYBR safe dye (Invitrogen, Paisely, UK). For whole-genome amplification: fragment C amplification was performed as for fragment B but with primers POLF1 and P5W in the first round and POLF2 and P4WRS in the second round. Finally, fragment A PCR employed primers P1 and P2 in the first round and P1 and P3 in the second round, with the same cycling conditions as for fragment B except for an annealing temperature of 55°C for the 20 cycles/30 cycles in round 1 and round 2, respectively.

The limit of detection of the fragment B (*pol* gene) assay was approximately 2.5 Log_10_ IU/ml. Nucleotide sequence was obtained for 194 specimens, although, a small subset (5.8%, n = 11) showed evidence of mixed infection in the *preS* region but not in the *S* gene and were thus included in the *S* gene mutation analysis but omitted from phylogenetic analysis. Consequently, 187 specimens were analysed for mutations in the *S* gene and 178 specimens were subgenotyped using phylogenetic methods. Genbank accession numbers for the *pol* gene sequences are JQ281112-JQ281258 and JQ281468-JQ281471.

Fragment D, encompassing the precore region, was amplified using the Qiagen HotStar Platinum *Taq*® (Qiagen, Crawley, UK) in both rounds of a nested PCR with the following cycling conditions for both rounds: denaturation at 95°C for 15 mins, 45 cycles of 94°C for 60 s, 50°C for 45 s an 72°C for 45 s and a final extension at 72°C for 10 mins [56]. Every assay contained negative and reagent only controls. The precore fragment was successfully amplified in 236 specimens; Genbank accession numbers JQ281259-JQ281467.

### TaqMan 5′ Nuclease Allelic Discrimination Assay

Genomic DNA from serum or plasma specimens from the virological analysis were genotyped for the *IL28B* SNP rs12979860 (n = 368) using the ABI 2× mastermix kit (Applied Biosystems) on the TaqMan 7300 platform (Applied Biosystems) as described previously [59].

### Statistical Analysis

Data is presented here as means ± standard deviation and ranges. Continuous variables, such as viral load, were compared between populations using the Student's t-test. Categorical data were analysed using Chi-squared and odds ratio tests. Associations between *IL28B* and HBeAg, confounding for age and sex, were examined using a linear regression analysis and SPSS software version 18.0. *p-*values<0.05 were considered statistically significant.

### Sequence Analysis and Phylogenetic Characterisation

For nucleotide sequence reactions, unincorporated primers and dNTPs were removed from amplified products using Exo-SAP IT (Affymetrix, Cleveland, USA), and subsequently sequenced bidirectionally on an ABI 3730 sequencing platform using primers detailed in Table S1.

In total, 178 fragments of 1,070 nucleotides of the HBV *pol* gene were compared with reference sequences available from Genbank representing each genotype and all subgenotypes of B and C. 236 sequences from the basal core promoter/precore fragment were analysed for mutations. The genotype of the 236 precore fragments was determined using online tools and BLAST analysis. Lasergene version 8 (DNASTAR, Madison, WI, USA) was used for contiguous sequence assembly [60], and the sequences were aligned using ClustalW [61], implemented in Bioedit version 7.05 [62]. A phylogenetic tree was constructed using the neighbour joining distance method under a Kimura-2-parameter model of evolution in PAUP* version 4.0 beta10 [63]. Statistical support for the topology of the trees was provided by 1000 bootstrap replicates. Reference sequences used represented all currently assigned genotypes of HBV, with the woolly monkey HBV strain (Genbank accession number: AF046996) used as an out-group. Numbering of HBV nucleotides starts at the *Eco*RI cleavage site or at homologous sites, if the *Eco*RI site is absent. HBV *pol* gene sequences were also analysed using the online genotyping tool Geno2Pheno (http://hbv.bioinf.mpi-inf.mpg.de/index.php) which compares the query sequence to reference sequences to identify known mutations associated with resistance to lamivudine, adefovir, entecavir, tenofovir and telbivudine, and with immune escape.

## Results

### Prevalence of HBV in Viet Nam

In total, 8654 specimens collected from eight well defined population groups in 5 different geographical regions were analysed for evidence of HIV, HBV and HCV infection. Of these, 10.7% (n = 925) tested positive for HBsAg, with a prevalence ranging from 9.4% in the lower risk groups (pregnant women, military recruits, patients admitted for elective surgery and blood donors) to a significantly elevated 17.4% (*p*<0.0001) in the IDU group (Figure 1). Notably, there was a high prevalence in the renal dialysis groups throughout the country (14.3%) and this was significantly higher than in the lower risk groups (*p*<0.0001).

10% of a subset of HBsAg positive samples further analysed tested positive for HBcIgM (n = 27/272), although many were weakly positive. Fifty percent of 110 HBsAg negative specimens, collected from all patient groups, had detectable anti-HBc demonstrating a significant number of resolved HBV infections. HBV DNA was undetectable in the 110 HBsAg negative specimens.

### Blood-Borne Virus Coinfections

In total, 58% of HBsAg positive IDUs and 22% of HBsAg positive CSWs had evidence of infection with at least one other blood-borne virus (Figure 2). Of 273 HBsAg positive specimens identified in the high risk study groups, coinfection with HIV was demonstrated in 28% of IDUs (n = 49/174; Figure 2) and 15.2% of CSWs (n = 15/99). Furthermore, HCV Ab/Ag was demonstrated in 89.8% of the HIV-HBV coinfections in IDUs (n = 44/49) and in 40% in CSW (n = 16/40). Evaluation of HBV-HIV coinfected patients revealed no significant differences in the mean HBV viral load or percentage of HBeAg negatives, compared to HBV monoinfected individuals (5.19 vs 5.23 Log_10_ IU/ml; 60% vs 59%).

10.7% (n = 34/318) of HBsAg positive specimens were positive for anti-HDV total antibody. The highest prevalence of total antibody was identified in IDUs (25.6%, n = 20/78; Figure 2), followed by military recruits (17.8%, n = 8/45), CSWs (8.8%, n = 5/57) and in a single dialysis patient (2.4%, n = 1/41). Anti-HDV total was not identified in any of the remaining low risk groups. Of note, 17.9% (n = 14/78) of HBSAg positive IDUs were quadruply infected with HBV-HIV-HCV-HDV. In HBV-HDV infected individuals, the mean HBV viral load was lower (4.2 Log_10_ IU/ml) and the seroconversion of HBeAg was higher (71%) when compared to HBV monoinfected individuals (5.2 Log_10_ IU/ml, 59% HBeAg negative, *p* = 0.194). 33% of 21 delta virus total antibody positive specimens were HDV IgM positive, all with low (<2.5 Log_10_ IU/ml) or undetectable HBV viral load.

### Molecular Analysis

73% (n = 273/376) of HBsAg specimens screened had detectable HBV viral load and the mean viral load was 5.21±2.56 Log_10_ IU/ml. Phylogenetic analysis showed that all individuals were infected with either genotype B or genotype C, and four distinct subgenotypes were identified (Figure 3). HBV subgenotype B4 (82.6%) predominated. Other genotypes detected included B2 (2.7%), C1 (14.6%) and C5 (0.5%). The newly described HBV putative “genotype I” was not detected.

The presence of HBeAg correlated significantly with viral load and the mean viral load in HBeAg positive samples was 7.58 Log_10_ IU/ml compared to 3.32 Log_10_ IU/ml in HBeAg negatives (*p*<0.0001; Table 1). Patients with HBeAg negative infection were on average significantly older (mean 32.7 vs 24.1 years; *p*<0.0001), had significantly lower or undetectable HBV DNA levels (DNA detected 65% vs 7%; *p*<0.01), and were more likely to be infected with genotype B (87.8% vs 79.7%; *p* = 0.29) than those who had HBeAg positive infection (Table 1).

### Genetic Variation within the Surface Antigen Region

187 HBV sequences were analysed for the presence of deletions and mutations in the surface gene. One or more point mutations were identified within the immunodominant ‘a’ region in 31% (n = 58/187) of samples analysed. Mutations associated with immune escape were identified in 23.5% (n = 45/187) samples and included the following residues Y100C (n = 6), T118K (n = 1), P120L/Q/S/T (n = 10), T123A/N (n = 2), I126N/S (n = 8), P127S (n = 2), Q129R (n = 2), G130D/N/R (n = 1), T131I (n = 4), M133L/T (n = 18), G144E (n = 1) and G145A/R (n = 4). Sixty-four specimens had mutations in the T cell epitope (including N40N/S, L42L/P, G44D/E/G, A45T, P46H, T47A/E/K/T). In the *preS2* region, 5.5% of specimens had a point mutation in the start codon which changed the amino acid to I (3.3%), V (1.7%) or T (0.55%), and 5.3% had a mutation at F22L with either a single or dual point mutation at this site.

Deletions were found in 5% (n = 9/187) of *S* gene sequences, ranging from 6 to 57 nucleotides in length. One sample had two deletions of 9 and 57 nucleotides in different regions of the *preS1* and *preS2* genes, and another sample had a deletion which involved the *preS2* start codon. The majority of these *S* gene deletions were identified in the genomes of high risk IDUs and CSWs (n = 8). No significant difference in history of vaccination was identified in individuals whose sequences contained mutations associated with immune escape (18.6%, n = 8/42) compared to sequences that did not (24%, n = 35/99; p = 0.431).

### Drug Resistance Mutations

Of 376 HBsAg positive specimens, only 19 (5%) were from individuals who reported having received treatment. All were coinfected with HIV and were receiving lamivudine as a component of HAART. 78.9% (n = 15/19) of these individuals had undetectable HBV viral loads and, of the 4 patients on HAART with a detectable HBV viral load, one – a HIV/HBV/HCV coinfected IDU – had drug resistance associated mutations, L180M and M204V in the *pol* gene conferring resistance to lamivudine and possible resistance to telbivudine. Analysis of other HBsAg positive and HIV Ag/Ab negative specimens (n = 165) did not reveal the presence of drug resistance associated mutations.

### Precore Mutations

Analysis of mutations in the basal core promoter and precore regions showed significant variation with genotype. 35% of all specimens had the precore stop mutation G1896A which abrogates HBeAg production; however, this was detected in 41% of genotype B compared to only 3% of genotype C viruses (*p*<0.0001; Figure 4). In contrast, the basal core promoter mutations A1762T and G1764A were detected more frequently in genotype C at 49% and 51% respectively, compared to only 22% and 18% in genotype B (*p*<0.0001). 1858C was detected only in genotype C viruses (n = 36). Evaluation of fragments for which there was an available corresponding *S* gene sequence also identified a difference in mutational patterns at the subgenotype level: B2 viruses compared to B4 for both G1896A (20%, n = 1/5 vs 40.2%, n = 53/132) and A1762T/G1764A (40%, n = 2/5 vs 19.7%, n = 26/132). We also identified a 21 bp deletion in the basal core promoter region of one sequence from a 55 year-old multi-transfused patient in Khanh Hoa (JQ281259).

### Genetic Variability at the IL28B Locus

HBsAg positive individuals (n = 368) from the five centres were genotyped for the *IL28B* SNP rs12979860 and the C allele frequency was found to be 93%. Overall, 86.41% (n = 318) were major homozygotes [CC], 13.04% (n = 48) were heterozygotes [CT] and 0.54% (n = 2) were minor homozygotes [TT]. A comparison of all monoinfected HBsAg positive individuals (n = 214) with individuals with resolved infection (i.e. HBsAg negative, HBc total positive and HBV DNA negative; n = 53) demonstrated no significant difference in genotype frequency (*p* = 0.50). Furthermore, HBV viral load was not significantly influenced by rs12979860 genotype (*p* = 0.24; Table 2) and multivariate regression analysis confounding for age and sex did not reveal a statistically significant association between the *IL28B* SNP and HBeAg positive and negative individuals (*p* = 0.06).

## Discussion

It is now clear that, as in many countries in Southeast Asia, HBV is endemic in Viet Nam and contributes to a significant burden of liver disease in the country. The present report provides one of the most comprehensive studies of HBV in Viet Nam to date, involving distinct geographical regions and populations with different risk factors for infection. The clinical outcome of HBV infection and the response to treatment and vaccination is influenced by a number of factors including coinfection with other blood-borne viruses, viral genotype, virus specific mutations in the pre-core and basal core promoter regions of the genome and a number of host factors. The present study has involved molecular analysis to analyse viral load, viral genotype and core and pre-core mutants. In addition our studies have examined coinfections with other blood-borne viruses including HIV, HCV and HDV – all of which would be expected to impact on HBV pathogenesis, and on the overall development and progression of liver disease.

The results of this study have confirmed the high level of endemicity of HBV in Viet Nam. As expected, levels of HBsAg are highest in the higher risk groups such as IDUs and CSWs, however, levels were also elevated in the dialysis cohorts from each region. Core IgM was detected in 27 samples and, while this may be suggestive of acute infection, confirmation is difficult due to the possibility of persistent detectable IgM in chronic infection. Serological analysis of HBsAg negative samples in eight groups in the study also demonstrated that some 50% of individuals tested had evidence of resolved HBV infection.

Our study also identified a very high prevalence of blood-borne viral coinfections. As expected, this was most evident in the IDU cohort, where serological analysis revealed that 53%, 28% and 26% of hepatitis B infected participants were coinfected with HCV, HIV and HDV, respectively. In fact, a significant proportion of these individuals had either triple or quadruple infections, with 18% of hepatitis B infected IDU patients infected with HIV, HCV and HDV. The prevalence of coinfection in the sex worker cohort, although marked, was less significant with 10%, 15% and 9% of the hepatitis B infected participants also infected with HCV, HIV and HDV, respectively. In contrast, there was little evidence of coinfections in the hepatitis B infected participants from lower risk groups.

High levels of HIV-HBV coinfection have significant implications for the introduction of HAART for HIV treatment. Our results suggest that it would be advisable that all HIV infected individuals in Viet Nam, if possible, should be screened for active HBV infection. A recent study from the US has shown that patients with chronic HBV, as defined by HBsAg positivity, had a much higher risk for developing the composite outcome of an AIDS-defining illness or death compared to individuals who were HBV negative [64]. Studies from South Africa have indicated that lamivudine, when employed as a sole HBV active drug in a HAART regimen, may not reliably suppress HBV replication [65]. As such, at least two drugs with anti-HBV activity (tenofovir plus lamivudine or plus emtricitabine) should be employed in the setting of HIV-HBV coinfection [66]. Indeed, tenofovir-based regimens are now being introduced as front line therapy of HIV infections in Viet Nam. This would also be expected to prevent the development of HBV resistance which can occur when single agents are employed, as we have found in the present study. Monitoring of such treatment should also, if possible be accompanied by measurement of both HIV and HBV viral loads to assess the efficacy of treatment. In addition to specific treatments, efforts should be enhanced to provide HBV vaccination to individuals with or at risk of HIV infection.

The present study also demonstrated significant levels of HDV infection. The overall prevalence across the cohorts of 10.7% (patients infected with HBV coinfected with HDV) is in a marked contrast to previous reports in Viet Nam, where rates of approximately 1% were described [11], [40]. The variation in HBV-HDV coinfection rates across the cohorts could be stratified between high and low risk exposure groups, with the vast majority of HDV infections occurring in the IDU and CSW cohorts. The difference between this and previous studies most likely reflects the inclusion of these defined high risk cohorts from urban centres. Molecular analysis of HBV DNA in HBV-HDV coinfected participants showed a significant reduction in detectable HBV viral load and a higher rate of e antigen negativity, when compared to the HBV monoinfected cohort. Our findings suggest that levels of HDV exposure in Viet Nam are significantly higher than previously recognised and that the burden of liver disease associated with HBV-HDV infection may be more substantial than previously thought.

Molecular studies demonstrated that HBV genotypes B4 and C1 predominated, but there were also smaller levels of B2 and C5. In agreement with a previous study, we did not identify the putative genotype I, suggesting that this recombinant remains rare in Viet Nam [46]. The lack of proofreading ability of the HBV polymerase, together with the high viral titre in active HBV infection, results in a high mutation rate during virus replication. Genetic mutations and deletions in the pre-S and basal core promoter regions of the HBV genome including T1753V, A1762T, G1764A, and C1766T have been associated with more severe liver disease and the development of HCC [67], [68]. The basal core promoter mutations 1762T/W and 1764A/R were detected in 26% and 24% of specimens, respectively. Previous reports of heterogeneity in the BCP/PC region of HBV in Viet Nam identified prevalence rates of up to 32% 1753C/A, 68% 1762T/1764A, 70% 1858C and 12% 1896A in genotype C viruses compared to 28% 1762T/1764A, 0% 1858C and 34% 1896A in genotype B [69], [70]. We identified the precore stop mutation G1896A in 35% (n = 82/236) of all samples, and this varied significantly with viral genotype, with a higher incidence in genotype B compared to genotype C, which is in agreement with previous studies [69], [70]. Contrastingly, the basal core promoter mutations 1762T and 1764A were detected more frequently in genotype C than in genotype B, and 1858C was identified only in genotype C viruses. Although the numbers were too low to reach statistical significance, we also identified a difference in the G1896A and A1762T/G1764A mutations at the subgenotype level, which has also been previously reported [71].

Of increasing concern is the emergence of mutations in epitopes of the HBV *S* gene which have been associated with immune escape [47]. As the HBV *pol* gene overlaps the *S* gene, mutations in the *pol* gene could result in non-synonymous mutations in the corresponding open reading frame. Thus, mutational pressure on the *pol* gene may potentially alter the antigenic characteristics of the surface protein, which could in turn alter vaccine efficacy and affect serological diagnostic assays targeting this region. We have found that 31% of our sequences contained mutations in the immunodominant region of the *S* gene and have identified a high number of potential immune escape mutants. Of particular note, 2.2% of sequences analysed contained the major vaccine escape mutation G145A as a dominant or as a mixed population. Analysis of the *preS* region demonstrated that a total of 5% of specimens had *preS* deletions (primarily identified in high risk individuals), 5.5% contained amino acid mutations in the start codon of the *preS2* and 5.3% had an F22L mutation in the *preS2*, all of which have been significantly associated with the development of HCC [68].

The role of host immunogenetics in influencing the rates of spontaneous HBV clearance and the development of persistent infection is poorly understood. *IL28B* genetic variants have previously been associated with natural and therapy-associated clearance of HCV [48], [72]–[74]. In contrast, recent studies have found no significant differences in *IL28B* allele frequencies between individuals who spontaneously clear or those who develop a persistent HBV infection [49], [50]. Interestingly, a Chinese study by Li and colleagues demonstrated an association between the rs12979860 CC genotype (associated with a favourable HCV response to therapy and higher natural clearance rates) with elevated IL28B protein levels in serum and with lower HBV viral loads (<10^5^ copies/ml) compared to both CT and TT genotypes. *IL28B* genotype has also recently been implicated in rates of HBeAg seroconversion (appearance of anti-HBe) following interferon-α treatment and also HBsAg clearance after follow up, although the latter effect was modest and further studies are required to corroborate these findings [51], [52]. In our study, comprising untreated, HBV monoinfected Vietnamese individuals, we did not observe a significant association between the *IL28B* genotype and clearance of HBsAg or variation in HBV DNA levels. Although we noted an association between *Il28B* and HBeAg status, this did not reach statistical significance which may be due to the low T allele frequency (7%) in Viet Nam. Our analysis does not exclude a role for this locus in influencing HBV viral replication and disease progression; however, our results suggest that, unlike the effect noted for spontaneous clearance of HCV, this host variant does not exert a strong influence on the course of HBV infection.

In conclusion, a number of studies including the present have clearly shown that Viet Nam is facing a huge burden of HBV related liver disease. Thus, the enhancement of HBV screening efforts in Viet Nam, in association with both vaccination and, if economically possible, treatment for chronic infection, should be prioritised to limit the future impact of serious liver disease. Moreover, in HIV infected individuals, testing for HBV should be implemented. Currently recommended treatment of coinfected individuals with a tenofovir-based regimen would provide effective therapy for both viruses and would be expected to prevent the emergence of HBV drug resistance mutations.

## Supporting Information

Table S1 **Primers used for the amplification and sequencing of HBV.**
(DOCX)
