# Supplementary material for: A Multicentre Molecular Analysis of Hepatitis B and Blood-Borne Virus Coinfections in Viet Nam
Source: PLoS One. 2012 Jun 13;7(6):e39027. doi: 10.1371/journal.pone.0039027 (PMC3374772; doi:10.1371/journal.pone.0039027)
Supplement: Table S1 — Primers used for the amplification and sequencing of HBV. (DOCX) [file pone.0039027.s001.docx]

**Table S1**

|  | **Primer** | **Nucleotide Sequence 5' - 3'** | **Direction** | **Position**  (relative to Ba, D00329) | **Reference** |
| --- | --- | --- | --- | --- | --- |
| ***Genotyping*** | | | | | |
| Fragment A | P1 | TTTTTCACCTCTGCCTAATCA | Sense | 1821-1841 | 54 |
|  | P2 | CCCTAGAAAATTGAGAGAAGTC | Antisense | 262-283 | 54 |
|  | P3 | CCACTGCATGGCCTGAGGATG | Antisense | 3193-3213 | 54 |
| Fragment B | P4mod | GCCTCATTTTGYGGGTCACCATA | Sense | 2801-2824 | 54† |
|  | P5mod | TTCKTTGACADACTTTCCA | Antisense | 979-997 | 54† |
|  | P6* | TTGGGGTGGAGCCCTCAGGCT | Sense | 3070-3090 | 54 |
| Fragment C | POLF1 | TATCGCTGGATGTGTCTG | Sense | 368-385 | ‡ |
|  | P5W | AAAAAGTTGCATGRTGMTGG | Antisense | 1805-1825 | 56† |
|  | POLF2 | CTGCTATGCCTCATCTTC | Sense | 416-433 | ‡ |
|  | P4WRS | CGCAGACCAATTTATGCCTAC | Antisense | 1782-1802 | 56† |
| Fragment D | PCF1 | TCTTGCCCAAGGTCTTACAT | Sense | 1636-1655 | 55 |
|  | PCR1 | AAACGAGAGTAACTCCACAG | Antisense | 1935-1953 | 55 |
|  | PCF2 | GGTCTTACATAAGAGGAC | Sense | 1646-1663 | 55 |
|  | PCR2 | TAACTCCACAGTAGCTCCA | Antisense | 1927-1945 | 55 |
| ***Sequencing*** | | | | | |
| Fragment A | P1 | TTTTTCACCTCTGCCTAATCA | Sense | 1821-1841 | 54 |
|  | P3 | CCACTGCATGGCCTGAGGATG | Antisense | 3193-3213 | 54 |
|  | T724 | GAACTCCCTCGCCTCGCAGAC | Sense | 2376–2396 | 57 |
|  | T725 | GATTGAGATCTTCTGCGACG | Antisense | 2414–2433 | 57 |
| Fragment B | P5mod | TTCKTTGACADACTTTCCA | Antisense | 979-997 | 54† |
|  | P6 | TTGGGGTGGAGCCCTCAGGCT | Sense | 3070-3090 | 54 |
|  | S2-2 | GGCACTAGTAAACTGAGCCA | Antisense | 668–687 | 57 |
|  | POLF1 | TATCGCTGGATGTGTCTG | Sense | 368-385 | ‡ |
| Fragment C | POLF2 | CTGCTATGCCTCATCTTC | Sense | 416-433 | ‡ |
|  | P4WRS | CGCAGACCAATTTATGCCTAC | Antisense | 1782-1802 | 58† |
|  | POLR1 | AGTATGGATCGGCAGAGG | Antisense | 1255-1272 | ‡ |
|  | S3 | GCTGACGCAACCCCCACTGG | Sense | 1186-1205 | 56 |
| Fragment D | PCF2 | GGTCTTACATAAGAGGAC | Sense | 1646-1663 | 57 |
|  | PCR2 | TAACTCCACAGTAGCTCCA | Antisense | 1927-1945 | 57 |

† modified with wobble bases

‡ Sequences provided by Dr. Pamela Cook, HPA, Antiviral Susceptibility Reference Unit, West Midlands Public Health Laboratory, Birmingham, UK
